# T2. DO ADVERSE LIFE EVENTS AT FIRST ONSET OF AUDITORY VERBAL HALLUCINATIONS INFLUENCE SUBSEQUENT VOICE-CHARACTERISTICS? RESULTS FROM AN EPIDEMIOLOGICAL STUDY

**DOI:** 10.1093/schbul/sby016.278

**Published:** 2018-04-01

**Authors:** Josef Bless, Frank Larøi, Julien Laloyaux, Kristiina Kompus, Bodil Kråkvik, Einar Vedul-Kjelsås, Anne Martha Kalhovde, Kenneth Hugdahl

**Affiliations:** 1 University of Bergen; 2 University of Bergen, University of Oslo, University of Liège; 3 University of Bergen, University of Oslo; 4 St. Olavs University Hospital; 5 St. Olavs University Hospital, Norwegian University of Science and Technology; 6 Jaeren District Psychiatric Center; 7 University of Bergen, University of Oslo, Haukeland University Hospital

## Abstract

**Background:**

Understanding what happens at first onset of auditory verbal hallucinations (AVHs) is important at both a clinical and theoretical level. Previous studies have focused on age with regard to first onset of AVHs. In the current epidemiological study, we investigated the role of adverse life events (e.g. accidents, divorce, bullying, unemployment) at the time of first onset of AVHs regarding symptom severity and general mental health later in life.

**Methods:**

Using data from the Launay-Slade Hallucination Scale (LSHS), we compared participants who reported having experienced at least one adverse life events at first onset of AHVs (Trigger group; N = 76) to those who did not report any specific events at first onset of AVHs (No-trigger group; N = 59) on a large array of variables using Fisher’s exact test.

**Results:**

Results revealed that the Trigger group experienced the AVHs as more emotional and they were also more troubled by the AVHs compared to the No-trigger group (all p < 0.01). Also, the Trigger group more often reported hallucinations in other (non-auditory) sensory modalities (e.g. visual, p = 0.012) compared to the No-trigger group. Furthermore, the Trigger group reported poorer mental health in general, and having had more frequent contact with mental health professionals, and also reported more frequently taking medication for mental problems in general (all p < 0.01).

**Discussion:**

Adverse life events at first onset of AVHs appear to have a negative influence on subsequent voice-characteristics and general mental health, suggesting their presence to be an important factor to take into account when determining the risk for psychosis or other mental disorders. However, future longitudinal studies are needed in order to corroborate these findings.

